# Identification of Key Residues in Dengue Virus NS1 Protein That Are Essential for Its Secretion

**DOI:** 10.3390/v15051102

**Published:** 2023-04-30

**Authors:** Brandon E. K. Tan, Michael R. Beard, Nicholas S. Eyre

**Affiliations:** 1 Research Centre of Infectious Diseases, School of Biological Sciences, University of Adelaide, Adelaide, SA 5005, Australia; 2 College of Medicine and Public Health (CMPH), Flinders University, Bedford Park, SA 5042, Australia

**Keywords:** dengue virus, NS1, NS1 secretion, mutagenesis, HiBiT, N-glycosylation

## Abstract

Dengue virus (DENV) non-structural protein 1 (NS1) is involved in multiple aspects of the DENV lifecycle. Importantly, it is secreted from infected cells as a hexameric lipoparticle that mediates vascular damage that is a hallmark of severe dengue. Although the secretion of NS1 is known to be important in DENV pathogenesis, the exact molecular features of NS1 that are required for its secretion from cells are not fully understood. In this study, we employed random point mutagenesis in the context of an NS1 expression vector encoding a C-terminal HiBiT luminescent peptide tag to identify residues within NS1 that are essential for its secretion. Using this approach, we identified 10 point mutations that corresponded with impaired NS1 secretion, with in silico analyses indicating that the majority of these mutations are located within the β-ladder domain. Additional studies on two of these mutants, V220D and A248V, revealed that they prevented viral RNA replication, while studies using a DENV NS1-NS5 viral polyprotein expression system demonstrated that these mutations resulted in a more reticular NS1 localisation pattern and failure to detect mature NS1 at its predicted molecular weight by Western blotting using a conformation-specific monoclonal antibody. Together, these studies demonstrate that the combination of a luminescent peptide tagged NS1 expression system with random point mutagenesis enables rapid identification of mutations that alter NS1 secretion. Two such mutations identified via this approach revealed residues that are essential for correct NS1 processing or maturation and viral RNA replication.

## 1. Introduction

Dengue virus (DENV) is a mosquito-borne (+) RNA virus of the Flavivirus genus within the *Flaviviridae* family. Epidemiological modelling has indicated that there are approximately 390 million DENV infections annually [1]. DENV infection is normally asymptomatic but can potentially lead to a wide range of clinical manifestations ranging from mild fever to severe forms of dengue, formerly categorized as dengue haemorrhagic fever (DHF) and life-threatening dengue shock syndrome (DSS) [2,3]. No specific antiviral therapies are currently available and the only licensed dengue vaccine available is only recommended for administration in individuals that are 9–45 years of age, and most importantly have at least one prior history of dengue infection [1,4]. Thus, there remains an urgent need for the development of safe and effective antiviral strategies, particularly in the form of vaccines and treatments, to effectively counteract the rise in dengue infection.

Following virus internalization into permissive host cells via clathrin-mediated endocytosis, the DENV nucleocapsid dissociates in the cytosol, liberating the positive-sense single-stranded RNA DENV genome, which is translated by host ribosomes at the rough endoplasmic reticulum (ER) [5]. The nascent 3400-amino-acid-long polyprotein is co- and post-translationally cleaved into three structural proteins (capsid, prM, and envelope) and seven non-structural proteins (NS1, NS2A, NS2B, NS3, NS4A, NS4B, and NS5) by host proteases and the viral DENV NS2B/NS3 protease complex [6]. The non-structural proteins promote endoplasmic reticulum (ER) membrane rearrangements, including the induction of disordered membranes, known as convoluted membranes (CMs), whose functions are unclear, and clusters of spherular invaginations of the ER, known as vesicle packets (VPs), which often remain connected to the cytoplasm via 10 nm pores and are the proposed sites of viral RNA replication [5,6,7,8,9]. Viral replication complexes (RC), constituting an incompletely defined collection of host cell factors, viral non-structural proteins and viral RNA, are anchored within these membranous compartments to enable efficient DENV replication mediated by the NS5 RNA-dependent RNA polymerase in a microenvironment that is shielded from host defence mechanisms [9,10]. The formation of viral nucleocapsids that contain newly synthesized viral RNA is proposed to occur in close proximity to viral replication sites and this is followed by nucleocapsid budding into envelope and prM enriched-ER membranes, acquiring a lipid envelope in the process. Virus assembly is thought to take place in close proximity to VPs, with assembled virions being stored in ER cisternae in ordered arrays encased by “virion bags” [9,11]. Individual virions are transported to the trans-Golgi network via secretory vesicles, where virion maturation occurs through cleavage of the prM protein by host resident protease, Furin, before the release of infectious mature viral particles via the conventional secretory pathway [6].

Flavivirus NS1 is a 48-kDa non-structural multi-functional glycoprotein that can be identified either as a membrane-associated (mNS1) species associated with vesicular compartments within the cell and the plasma membrane or a secreted extracellular NS1 species (sNS1) [12,13,14]. Intracellular mNS1 is essential in virus replication and has been observed to co-localize with components of viral replication complexes in mammalian cells and insect cells [7,15,16,17,18,19,20,21,22]. The sNS1 is a soluble lipoprotein particle forming an open-barrel hexamer exterior, with its central internal channel tightly packed with lipid components resembling those of plasma high-density lipoproteins (HDLs) that are involved in systemic lipid traffic and vascular homeostasis [23]. Interestingly, a recent cryo-EM study has indicated that NS1 hexamers interact with and collapse upon HDLs, and that this interaction of sNS1 with lipoproteins is important for the ability of sNS1 to induce inflammatory cytokine production by macrophages [24]. Another high-resolution cryo-EM study has also recently demonstrated that, in addition to hexamers, the majority of sNS1 is found in either ‘loose’ or ‘stable’ tetrameric forms, which are differentially susceptible to the antibody mediated blockade of vascular damage [25]. The DENV NS1 dimer 3D structure has been successfully resolved, revealing that NS1 comprises three main domains: a small N-terminal β-roll domain (amino acids position 1 to 29) formed by disulphide linkage of two β-hairpins, the Wing domain (amino acids position 38–151) flanked by connector subdomains (amino acids position 30–37 and 152–180), and finally, a core β-ladder domain (amino acids position 181–352) that stretches along the whole length of the dimer via its 18-antiparallel β-strands that are arranged like rungs of a ladder [22,26,27]. The β-roll domain and the connector subdomain of the Wing domain form a protrusion that causes one face of the dimer to be hydrophobic such that is thought to mediate the association of the NS1 dimer with the inner leaflet of the ER membrane. In its hexameric form, three β-rolls face the interior of the hexamer, interacting with the central lipid cargo. In both mNS1 and sNS1 forms, the distal tips of the β-ladder and the Wing domain disorientated loops contain N-glycosylation sites (amino acids position 130 and 207) that protrude outwards, enabling them to be exposed to the external environment.

Following the cleavage of the NS1-NS2A junction by an undefined protease in the ER, glycosylation of DENV NS1 occurs whereby high mannose carbohydrate moieties are added to asparagine residues 130 and 207 [12,28]. In the Golgi, additional NS1 glycan modification occurs, where a complex-type sugar is incorporated onto the N130 glycan, while the high mannose moiety on N207 remains [28]. N-glycosylation on NS1 has been demonstrated to be essential in many NS1 functions throughout the DENV life cycle. Mutating either the N130 or N207 glycosylation site within DENV NS1 appeared to impede virus growth, thus reducing DENV neurovirulence in a mouse model [29]. The alteration of both N-linked NS1 glycosylation sites interestingly resulted in the generation of relatively unstable mutant viruses, indicating that the glycosylation of these sites is important for viral replication. Additionally, it was demonstrated that the DENV NS1 N130 glycan is essential for the stabilisation of secreted NS1, whereas the N207 glycan promotes the stability of the extracellular NS1 protein and its secretion [30]. Recently, a related study by Wang et al. (2019) indicated that the DENV NS1 N130 glycan is essential for NS1 protein secretion, whereas the N207 glycan is dispensable for both NS1 stability and secretion, contrasting with the results from the earlier study by Somnuke et al. 2011 [30,31]. Regardless, these N-glycans appeared to be intimately involved in NS1 secretion and the stability of the NS1 dimer.

sNS1 has been strongly associated with the pathogenesis of severe DENV infections since early studies reported its detection at significantly elevated levels in the serum of DENV-infected patients [32,33,34,35]. Highly immunogenic sNS1 and plasma-membrane-associated NS1 are known to be essential in immune evasion through a range of mechanisms, including the activation or inhibition of complement pathways and the elicitation of autoantibodies that cross-react with platelets and endothelial cells leading to endothelial dysfunction; one of the distinctive hallmarks of flavivirus disease pathogenesis [14,35,36,37,38,39].

In addition to its roles in triggering endothelial permeability via complement and auto-antibodies, sNS1 has also been recognized as a pathogen-associated molecular pattern (PAMP) that promotes the release of vasoactive chemokines and cytokines from human peripheral blood mononuclear cells (PBMCs) via TLR-4 activation, subsequently leading to vascular leakage [40,41]. Importantly, pre-treatment of endothelial cells with anti-TLR-4 antibody effectively inhibited sNS1-mediated vascular leakage, further demonstrating that sNS1-mediated TLR-4 activation in endothelial cells directly contributes to vascular leakage. In addition, sNS1 has been shown to promote the disruption of the endothelial glycocalyx-like layer (EGL) of human pulmonary endothelial cells, which are responsible for endothelial barrier function. The sNS1-mediated activation of cellular enzymes, including heparinase, sialidases and cathepsin L, has been shown to induce the degradation and shedding of components of the EGL-layer, subsequently leading to the disruption of its integrity and endothelial hyperpermeability [42]. More recently, a cohort of flavivirus NS1 proteins, including DENV NS1, was examined for their ability to induce endothelial barrier dysfunction in vitro and hyperpermeability in vivo in a tissue-specific manner, thus reflecting the unique pathogenesis, disease, and dissemination of respective flavivirus infections [43].

Several recent comprehensive mutagenesis studies have investigated which NS1 residues are critical to its roles in viral RNA replication, infectious virus particle production, NS1 secretion, and the pathogenic effects of sNS1 [22,44,45]. Despite this and the recognized importance of NS1 secretion to DENV disease pathogenesis, the molecular features of NS1 that are important to its secretion remain incompletely understood. In this study, we have employed random mutagenesis of NS1 in combination with the HiBiT luminescent peptide tagging system to identify mutations that disrupt its secretion activity from transfected cells. This led to the identification of 10 point mutations that resulted in markedly impaired NS1 secretion, with most of these mutations mapping to the β-ladder domain. Subsequent analyses of these mutants by confocal microscopy revealed similar localization patterns to that of the wildtype NS1, while initial Western blotting indicated that many of these mutant NS1 proteins may be inherently unstable and/or improperly folded. In support of this interpretation, repetition of these Western blotting experiments using an anti-HiBiT tag antibody revealed ready detection of all of the mutant NS1-HiBiT proteins and enabled confirmation of the secretion-impaired phenotypes of these mutants. Follow-up studies of two of these mutants, V220D and A248V, revealed that they prevented viral RNA replication and, when expressed in the context of an NS1-NS5 polyprotein, exhibited an ER-like localization pattern and were once again not detectable by Western blotting, implying that these mutant NS1 proteins may prevent viral RNA replication as a result of failing to mature properly.

## 2. Materials and Methods

### 2.1. Cell Culture, Antibodies, Conjugates, and Fluorescent Dyes

The Huh-7.5 human hepatocellular carcinoma cell line was kindly provided by Charles M. Rice (Rockefeller University, New York, NY, USA). Human embryonic kidney-derived 293FT cells were purchased from Thermo Fisher Scientific. Huh-7.5 cells stably expressing T7 RNA polymerase (Huh-7.5 + T7RNA pol) have been previously described [46]. All of these cell lines were cultured and maintained in a 37 °C, 5% CO_2_ humidifier incubator in complete Dulbecco’s modified Eagle medium (DMEM) with HEPES (Invitrogen, USA) supplemented with 1% penicillin-streptomycin (Sigma) and 10% foetal bovine serum (FBS), referred to henceforth as complete DMEM. Huh-7.5 + T7 RNApol cells were cultured, as above, with puromycin (Sigma-Aldrich) added to a final concentration of 3 µg/mL.

Mouse anti-flavivirus NS1 protein ‘4G4′ monoclonal antibody was kindly provided by Dr Jody Peters and Prof. Roy Hall (University of Queensland, Australia) or purchased from Mozzy Mabs (University of Queensland, Australia). Mouse monoclonal antibody recognizing β-actin (AC-15) was purchased from Sigma-Aldrich. Mouse anti-NS3 monoclonal antibody (GT2811) was purchased from GeneTex, USA. Mouse anti-HiBiT monoclonal antibody (30E5) was purchased from Promega. Mouse anti-capsid monoclonal antibody ‘6F3.1′ was kindly obtained from Professor John G. Aaskov (Queensland University of Technology, Australia). IRDye^®^ 800 CW goat anti-mouse IgG secondary antibody for Western Immunoblotting was purchased from LI-COR Biosciences, USA). AlexaFluor 488- and 555-conjugated anti-mouse IgG and anti-rabbit IgG secondary antibodies were purchased from Thermo Fisher Scientific.

Fluorescent stain 4′,6-diamidino-2-phenylindole (DAPI) was purchased from Sigma-Aldrich. Double-stranded DNA dye, DRAQ5^®^, was purchased from Thermo Fisher Scientific. A cell permeant stain that labels the endoplasmic reticulum, IraZolve-ER Blue^®^, was purchased from REZOLVE Scientific (Australia) or kindly provided by Dr Sally Plush (UniSA, Adelaide, Australia). Additional details about antibodies and dyes, including catalogue numbers, are detailed in Supplementary Table S1.

### 2.2. Cloning of Plasmid DNA Constructs

A synthetic version of the full length DENV-2 strain 16681 (pFK-DVs), a derivative full-length reporter construct that encodes a *Renilla* luciferase reporter gene (R2A) (pFK-DVs-R2A) and a *Renilla* luciferase-encoding sub-genomic replicon construct (pFK-sgDVs-R2A) were kindly provided by Prof. Ralf Bartenschlager (University of Heidelberg, Germany) [47]. For site-directed mutagenesis (N130A, N207A, V220D, A248V), external primers (*Mlu*I ext FWD and *Kas*I ext REV) were used in combination with internal mutation-encoding primers (see Supplementary Table S2 for oligonucleotide sequences) to generate two PCR-derived fragments featuring 10–20 bp overlaps using Q5 DNA polymerase (New England Biolabs). These fragments were gel-purified before being assembled into *Mlu*I/*Kas*I-digested pFK-DVs plasmid based on a 2-fragment Gibson Assembly reaction using NEBuilder^®^HiFi DNA Assembly (New England Biolabs). Sequences of manipulated DNA regions within plasmids were confirmed by automated DNA Sanger sequencing (Australian Genome Research Facility; AGRF, Australia).

### 2.3. Generation of DENV-2 NS1 Expression Constructs with Point Mutation (s)

To generate a library of NS1 expression plasmids featuring 1 point mutation in each clone on average, PCR-based random point mutagenesis using the GeneMorphII Random Mutagenesis Kit (Agilent Technologies) was carried out using a forward primer encoding an N-terminal start codon and the first 6 codons of the C-terminal of the envelope (E) protein coding region (pLenti6 NS1 FWD) and a reverse primer encoding a C-terminal HiBiT tag and stop codon (pLenti6 NS1 REV) (see Supplementary Table S2 for oligonucleotide sequences). PCR-based random point mutagenesis reactions were set up in 50 μL volumes containing 150 ng of pFK-DVs plasmid DNA as template, 250 ng of each forward/reverse primer and dNTPs, reaction buffer and DNA polymerase as per the manufacturer’s recommendations. Samples were amplified via 30 cycles of PCR as per the manufacturer’s instructions. PCR products were *Dpn*I-treated for 2 h at 37 °C and gel-purified before cloning into a *Bam*HI/*Xho*I digested lentiviral expression plasmid construct (pLenti6-V5-D-TOPO) (Thermo Fisher, USA) using NEBuilder^®^ HiFi DNA Assembly (NEB). Colony PCR was performed to verify the presence of potentially mutagenized NS1 cDNA before proceeding to subsequent HiBiT luciferase reporter-based assays (Promega). Out of 300 bacterial colonies randomly selected for colony PCR, 173 clones were confirmed to possess a potentially mutagenized NS1 insert.

### 2.4. HiBiT Luciferase Reporter-Based Assays

Huh-7.5 cells were seeded into 24-well plates and cultured overnight before transient transfection with purified uncharacterized NS1 expression clones using Lipofectamine 2000 (Thermo Fisher Scientific), according to the manufacturer’s instructions. At 48 h post-transfection, supernatant samples were collected, cleared by centrifugation and mixed with an equal volume of 2X Passive Lysis Buffer (Promega). 1X Passive Lysis Buffer was added to the remaining transfected cell monolayers, pipetting (10×) before harvesting the lysate samples. Supernatant and lysates of respective samples were then probed for their luciferase signals using a Nano-Glo HiBiT Lytic Detection System (Promega) and a GloMax^®^ Discover Microplate Reader (Promega) as per the manufacturer’s instructions.

### 2.5. SDS-PAGE and Western Blotting

Supernatant and cell lysate samples were harvested and prepared from transfected Huh-7.5/293FT cells using NP-40 lysis buffer (1% NP-40, 50 mM Tris-HCl [pH 8], 150 mM NaCl) containing protease inhibitor cocktail (Sigma-Aldrich), as described previously [46]. Samples were mixed with non-reducing sample buffer for anti-NS1 (mAb 4G4) Western blotting or reducing sample buffer for Western blotting using all other antibodies, boiled for 5 min at 95 °C, separated via SDS-PAGE and transferred to nitrocellulose membranes (Bio-Rad). Following blocking for 1 h using 5% (*w*/*v*) skim milk in TBS, membranes were incubated with anti-NS1 primary antibody (1:10) (See Supplementary Table S2 key reagent resources) in TBS containing 0.1% (*v*/*v*) Tween-20 (Sigma-Aldrich) and 1% (*w*/*v*) skim milk at 4 °C overnight. Following stringent washing, membranes were incubated with IRDye^®^ 800 CW goat anti-mouse IgG secondary antibody (1:10,000) for an hour in the dark at room temperature (RT) before being washed and imaged using a LI-COR Odyssey imaging system (Flinders Proteomics Facility, Flinders University, Australia).

### 2.6. Immunofluorescence Analyses

Huh-7.5 cells were seeded into 8-well coverslip bottomed chamber slides (ibidi Gmbh, Germany) that were pre-coated with 0.2% (*w*/*v*) gelatin. Following overnight culture, cells were transiently transfected with NS1 expression plasmid constructs of interest using Lipofectamine 3000 (Thermo Fisher Scientific), according to the manufacturer’s instructions. At 24–30 h post-transfection, cells were fixed with ice-cold acetone: methanol (1:1) fixative for 15 min at 4 °C, washed once with PBS and blocked with 5% (*w*/*v*) BSA in PBS at RT for 30 min.

For DENV NS1 staining, mouse anti-NS1 monoclonal antibody ‘4G4’ was utilized in combination with goat anti-mouse IgG AlexaFluor 488 conjugated secondary antibody (Thermo Fisher Scientific). Nuclear DNA was stained with DAPI dye (Sigma Aldrich) diluted to 1 µg/mL in PBS for 10 min in the dark. Alternatively, nuclei were stained with DRAQ5 (Invitrogen) by incubating fixed samples for 15 min at RT with 0.5 µM DRAQ5 in PBS. IRaZolve-ER Blue^®^ dye (REZOLVE Scientific, Australia) was used for endoplasmic reticulum staining by incubating fixed samples in the dark at RT with IraZolve-ER at 25 µM in PBS. The samples were then washed with PBS prior to imaging. Z-stacks of respective samples (with 0.2 µm step-size) were acquired using a ZEISS LSM 880 Fast Airyscan confocal fluorescence microscope system using a C Plan-Apochromat 63X oil immersion objective (NA:1.4) and 2.5× zoom (Flinders Microscopy and Microanalysis, Flinders University, Australia). Appropriate laser lines (405, 488, 561 and 633 nm) were used at 2% of maximal power, adjusting master gain settings to enable signal visualisation with minimal saturation. Pinhole sizes were set to 1.0 Airy units for the longest-wavelength fluorophore and matched for all tracks. Images were acquired at 1024 × 1024 pixels, with an xy pixel size of 50 nm. The images were processed and analysed using ZEN Blue (version 3.2) software (ZEISS). Colocalization analysis was performed by measurement of Pearson’s correlation coefficients for each cell (>20 cells/group) following drawing Bezier regions of interest around each cell using ZEN Blue. Highlighting of colocalized pixels was performed using the ‘Colocalization’ tool of ZEN Blue following setting conservative thresholds that were consistent across all images for a given experiment.

### 2.7. Multiple Sequence Alignments and In Silico Analyses

Sequence alignments of NS1 protein sequences were performed using Jalview software ver.2.11.1.4, with the following isolates (UniprotKB [Swiss-Prot accession no.]), as detailed: DENV-1 Brazil/97011/1997 strain (P27909); DENV-2 Thailand/16681-PDK-53 strain (P29991); DENV-3 Martinique/1243/1999 strain (Q6YMS3); DENV-4 Thailand/0348/1991 strain (Q2YHF0); West Nile virus (P06935), and Yellow Fever virus Ivory Coast/1999 (Q6J3PI) using the in-built ClustalW scoring algorithm. In silico analyses were conducted using the UCSF^®^ Chimera software ver.1.13.1. on the established DENV-2 NS1 crystal structure derived from Protein Data Bank accession 4O6B.

### 2.8. In Vitro RNA Transcription and RNA Transfection

A total of 5 µg of plasmid DNA of interest was linearized overnight at 37 °C with *Xba*I before being purified using a Nucleospin^®^ Gel and PCR Clean-up kit (Macherey-Nagel). In vitro RNA transcription was carried out via SP6 RNA polymerase reactions using a mMessage mMachine^®^ Kit (Thermo Fisher Scientific) as per the manufacturer’s instructions. Samples were treated with 1 μL of TURBO Dnase (Thermo Fisher Scientific) before proceeding to RNA isolation. In vitro RNA transcripts were purified using TRIreagent (Sigma-Aldrich), as per the manufacturer’s instructions, and RNA pellets were resuspended in nuclease free water. The integrity of RNA transcripts was validated by agarose gel electrophoresis. For RNA transfection, cells were transfected with RNA transcripts using DMRIE-C transfection reagent (Thermo Fisher Scientific) in reduced serum Opti-MEM medium (Thermo Fisher Scientific) as per the manufacturer’s instructions. The transfection medium was replaced by complete DMEM at 3–4 h post-transfection.

### 2.9. Infectivity Assays

Prior to conducting focus-forming unit (FFU) assays, virus-containing supernatants were collected at respective timepoints, cleared of cells and debris by centrifugation, and stored at −80 °C. To determine the viral titers, Huh-7.5 target cells were seeded into 96-wells plates at 2 × 10^4^ cells/well a day prior to the infection. The cells were inoculated with 40 µL/well of serially diluted virus-containing supernatants. The cells were then cultured for 3 h before washing with PBS and returning to culture for 72 h. At 72 h post-infection, the cells were fixed with ice-cold acetone: methanol (1:1) for 15 min at 4 °C. The infected cell foci were enumerated following the indirect immunofluorescent labelling of the DENV capsid protein using the mouse anti-capsid monoclonal antibody (6F3.1, diluted 1:5 in 1% BSA/PBS) and the conjugated secondary antibody (Alexa Fluor 488, diluted 1:400 in 1% BSA/PBS). Virus infectivity was expressed as focus-forming units (FFU)/mL.

### 2.10. Graphics and Statistical Analyses

Several diagrams were created using the online tool platform, BioRender^®^. All graphs were generated using GraphPad Prism 9 software.

## 3. Results

### 3.1. Identification of DENV-2 NS1 Residues That Are Essential for Its Secretion via Random Point Mutagenesis and Luminescent Peptide Reporter Assays

**Figure 1:**
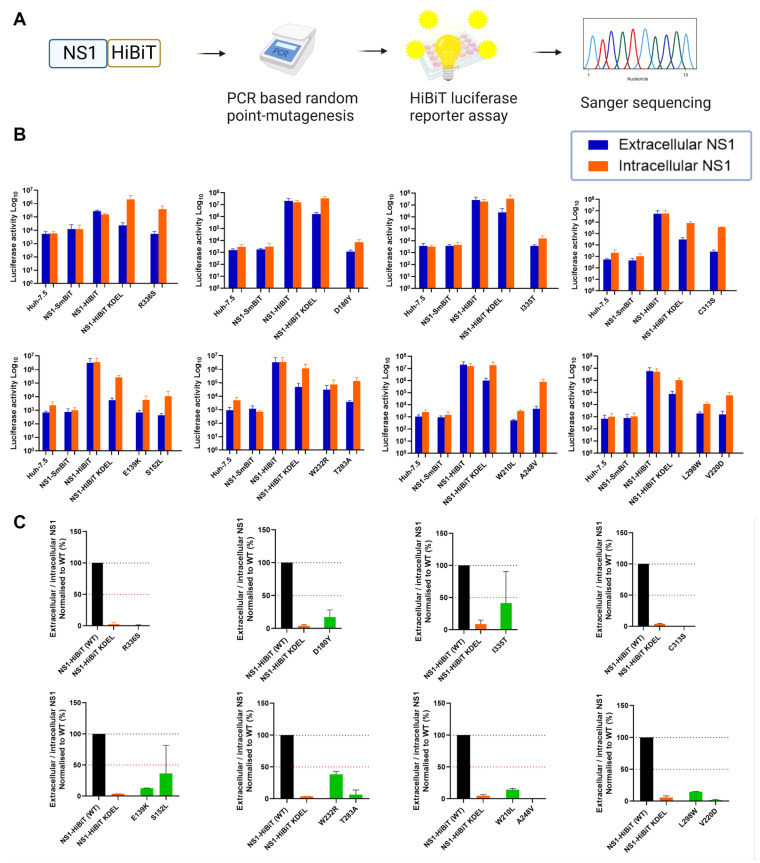
Identification of NS1 mutant clones with impaired NS1 protein secretion. (**A**) Schematic diagram of the experimental approach. Wild-type DENV-2 NS1 was subjected to PCR-based random point mutagenesis before incorporating the mutagenized NS1 into an expression plasmid construct containing the C-terminal HiBiT tag. Seeded Huh-7.5 cells were transiently transfected with the indicated NS1 expression plasmids, and supernatants and lysates were harvested at 48 h post-transfection. (**B**) Supernatants and lysates were collected from cells that were transfected with NS1-SmBiT (negative control), NS1-HiBiT (wildtype), NS1-HiBiT KDEL (positive control for intracellular NS1 retention) or mutagenized NS1-HiBiT expression clones, as indicated, and probed for their luciferase activity via the Nano-Glo^®^ HiBiT Lytic Detection System. Orange bars represent intracellular (lysate) samples, while blue bars represent extracellular (supernatant) samples. Data for mutagenized clones are only depicted for those clones that encoded a single amino acid substitution and displayed a >50% reduction in the ratio of the extracellular-to-intracellular luciferase activity. Data are presented as raw luciferase activity (relative light units [RLU]) means ± SD of duplicates for each group, from two independent experiments. (**C**) Extracellular-to-intracellular NS1 luminescence ratios were determined for each sample in (**B**), and expressed as a percentage of wildtype NS1-HiBiT values. Black bars represent wildtype NS1-HiBiT, orange bars represent NS1-HiBiT KDEL, while green bars represent NS1-HiBiT point mutants, as indicated. A black dotted line is indicated at 100%, while a red dotted line is indicated at 50%. Data are means ± SD of duplicates for each group from two independent experiments.

A recent study identified a cluster of several highly conserved amino acid residues within the β-ladder domain of NS1, which are essential for NS1 secretion [44]. To further elucidate the molecular determinants of NS1 that are essential for its secretion, we generated a library of expression plasmid constructs encoding random NS1 point mutations and bearing a C-terminal HiBiT luminescent peptide tag. To screen for NS1 mutations that impact NS1 secretion, NS1 expression clones that resulted from PCR-based random point mutagenesis were transfected into cells prior to the measurement of extracellular and intracellular NS1-associated luminescence activities (Figure 1A). In this system, HiBiT-tagged proteins can be readily detected with the addition of the complementary binding partner “LgBiT”, which auto-associates with HiBiT-tagged proteins to generate a strong luminescence signal [48]. Thus, the luminescence signal generated is directly proportional to the amount of the HiBiT-tagged protein in the samples.

We prepared a collection of 173 potentially mutagenised NS1-HiBiT expression constructs, and an analysis of these constructs by HiBiT luciferase assays revealed numerous mutagenised NS1 expression clones with altered ratios of extracellular-to-intracellular NS1 luminescence activities (Figure 1B). As many of these constructs contained multiple mutations that complicated interpretations, only clones with a single coding point mutation and a >50% decrease in extracellular-to-intracellular luciferase activity ratios are depicted. Wildtype NS1-HiBiT generated an almost equivalent luciferase activity in intracellular and extracellular samples across each independent luciferase experiment. The NS1-SmBiT construct (negative control) yielded negligible levels of luminescence, which were similar to those of untransfected parental Huh-7.5 cells, consistent with the SmBiT peptide’s weak association with the LgBiT protein [48,49]. An NS1-HiBiT construct with an ER-retention motif, KDEL, displayed intracellular NS1 luminescence activity that was markedly higher than that of the corresponding extracellular NS1 samples. This indicated that the KDEL motif attached to the carboxy terminus of the NS1-mediated ER retention of the NS1-HiBiT protein, as anticipated, supporting its utility as a positive control for the inhibition of NS1 secretion. Extracellular-to-intracellular NS1 luminescence ratios were subsequently determined for each NS1 clone to identify clones bearing mutation(s) that impact NS1 secretion activity (Figure 1C). Several NS1 mutant clones displayed a greater than 50% decrease in NS1 secretion efficiency, and were submitted for Sanger sequencing.

Sanger sequencing revealed varying mutation frequencies among these NS1 mutant clones. To simplify interpretations, only those NS1 clones bearing single coding mutation(s) were considered for subsequent experiments. Accordingly, the identified mutants, E139K, S152L, D180Y, V220D, A248V, T283A, L298W, C313S, I335T, and R336S, were selected for in silico analyses. Although W210L and W232R were shown to exhibit a greater than 50% decrease in NS1 secretion efficiency, they were excluded from this study, given that their luminescence activities closely resembled those of the wild-type NS1-HiBiT-expressing cells and parental Huh-7.5 cells, respectively.

### 3.2. In Silico Analyses Indicate That the Majority of NS1 Secretion-Impairing Mutations Are Located within the Carboxy-Terminal Region of the β-Ladder

**Figure 2:**
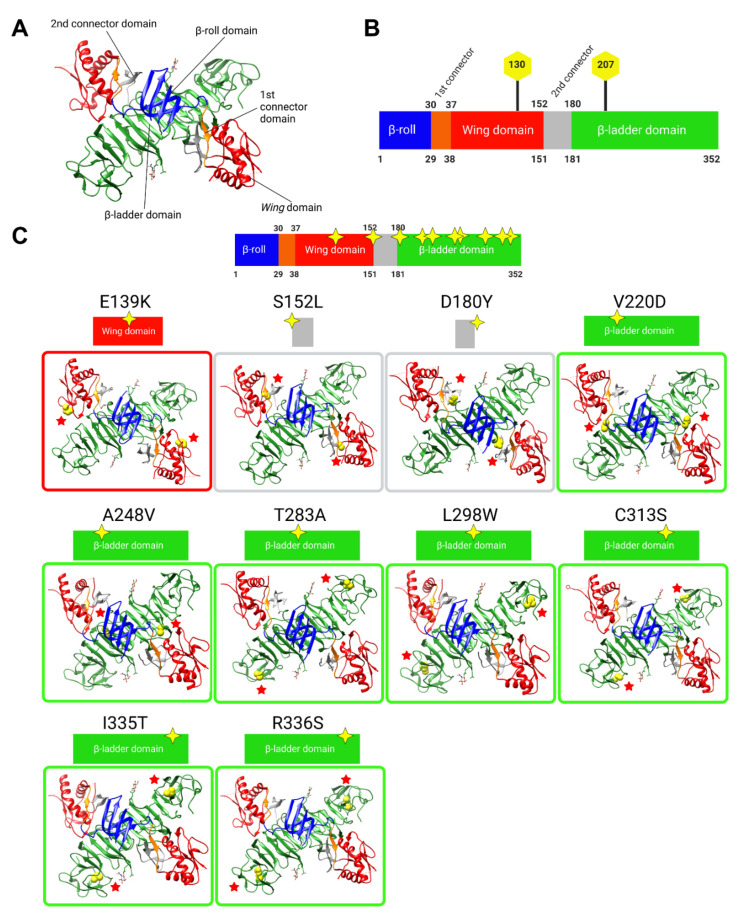
In silico analyses of the identified NS1 secretion-impaired mutants. (**A**) Three-dimensional structure of the NS1 dimer. (**B**) Schematic diagram representation of NS1 protein domains, which have been color-coded to match those of the 3D dimer structure in (**A**). The residue locations of junctions between domains and N-glycosylation sites are indicated. (**C**) Overall in silico analyses of the 10 selected NS1 secretion-impaired mutants identified from Figure 1. Homology model of the 3D structure of NS1 dimer retrieved from Protein Data Bank entry 4O6B, with mutated residues portrayed as van der Waals spheres in yellow and indicated with red stars for easier visualisation. From left to right, the β-roll, 1st connector, Wing, 2nd connector, and β-ladder are highlighted in blue, orange, red, grey and green, respectively (**A**), and this color-coding is also maintained in the diagrams in (**B**) and graphics (**C**). Yellow stars indicate the approximate amino acid positions in each domain. In silico analyses were carried out using the UCSF^®^ Chimera software ver.1.13.1.

**Figure 3:**
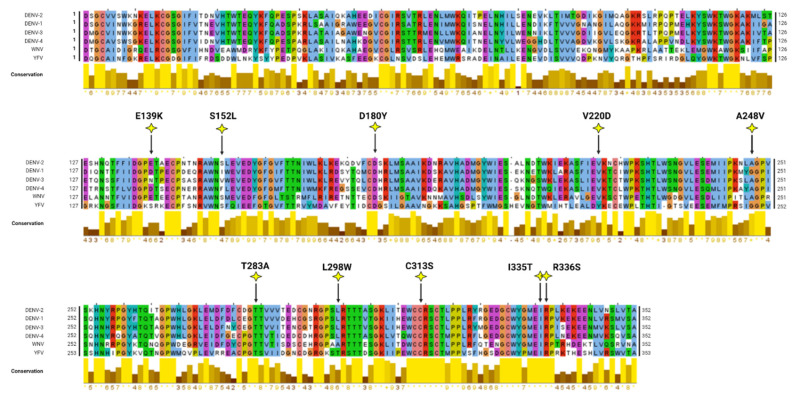
Mutiple sequence alignment of various NS1 proteins. Alignment of NS1 amino acid sequences of different DENV serotypes and two related flaviviruses (West Nile virus [WNV], Yellow Fever virus [YFV]) was performed using the in-built ClustalW algorithm in Jalview. The graphical displays below the alignments show the relative amino acid conservation across the various NS1 sequences with yellow, indicating the highest degree of conservation across the sequences. The positions of the mutated residues are indicated as yellow stars within the NS1 amino acid sequence alignment.

In silico modelling analyses were conducted to map and visualise the locations of NS1 secretion-impairing mutations on the established NS1 dimer crystal structure (Figure 2A,B). Interestingly, most of the NS1 secretion-impairing mutations identified in this study localised to the β-ladder domain (V220D, A248V, T283A, L298W, C313S, I335T, R336S), while two mutations flanking the second connector sub-domain (S152L and D180Y) interconnecting the Wing domain and β-ladder domain and one mutation within the Wing domain (E139K) were also identified (Figure 2C). Additionally, an alignment of NS1 amino acid residues of all DENV serotypes, as well as those of the West Nile virus (WNV) and Yellow Fever virus (YFV), was conducted, revealing the position of each NS1 secretion-impairing mutation and their relative conservation across various NS1 protein sequences (Figure 3).

### 3.3. Western Blot Analysis of Putative Secretion-Impairing NS1 Mutations

**Figure 4:**
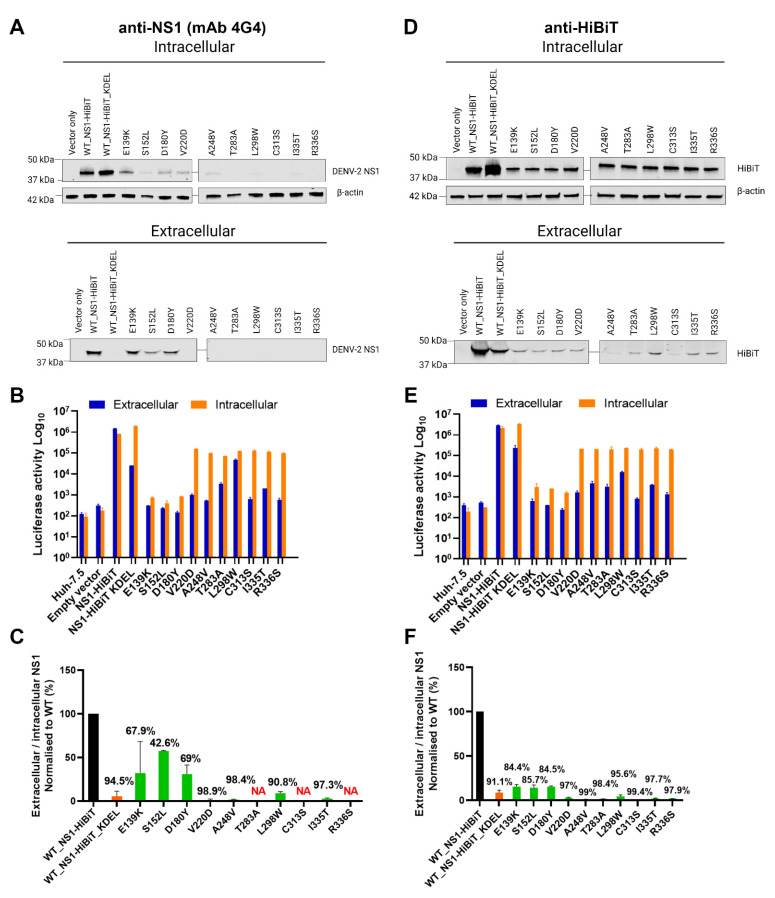
Western blot analysis of NS1 protein secretion efficiencies for putative NS1 secretion-defective mutants. (**A**,**D**) At 48 h post-transfection, supernatants (‘extracellular’) and cell lysates (‘intracellular’) were harvested from Huh-7.5 cells that were transfected with the indicated expression constructs, before subjecting samples to SDS-PAGE and Western blotting to detect DENV NS1 using a conformation-specific anti-NS1 antibody (**A**) or an anti-HiBiT tag antibody (**D**). β-actin was also detected in parallel as a loading control for lysate samples (**A**,**D**). (**B**,**E**) Lysate and supernatant samples that were used in Western blotting (**A**,**D**) were also analysed via HiBiT luciferase assays (**B**,**E**). Orange bars represent intracellular (lysate) samples, while blue bars represent extracellular (supernatant) samples. (**C**,**F**) Quantitative analysis of Western blotting data obtained using anti-NS1 and anti-HiBiT antibodies (**A**,**D**, respectively). Extracellular-to-intracellular NS1-HiBiT mutant protein levels were expressed as a percentage of values for the respective wildtype control samples. Black bars represent wildtype NS1-HiBiT, orange bars represent NS1-HiBiT KDEL, while green bars represent NS1-HiBiT point mutants, as indicated. Data are means ± SD from two independent replicates, with the values above each error bar indicating %-decrease in NS1 secretion efficiency compared to wildtype values. Note that NS1 mutants, T283A, C313S, and R336S were excluded from quantification analyses in (**C**) (NA; not assessed), given the inability to detect them by Western blotting (**A**).

Given that the our identification of NS1 secretion-impairing mutants was exclusively based on luciferase readout values (Figure 1), we further investigated the impairment of NS1 secretion activity by these NS1 mutants via Western blotting using a conformation-specific anti-NS1 antibody (mAb 4G4) (Figure 4A). Intracellular DENV-2 NS1 was strongly detected for wildtype (WT) NS1-HiBiT and KDEL-tagged NS1-HiBiT in cell lysates, while E139K, D180Y, and V220D NS1-HiBiT mutants were also readily detected in transfected cell lysates. Other NS1 mutants, including S152L, A248V, L298W, and I335T, appeared to be weakly expressed, while T283A, C313S, and R336S NS1 mutants could not be detected by Western blotting using this antibody. The extracellular DENV-2 NS1 protein was only detected in WT, E139K, S152L, and D180Y NS1 supernatant samples, while, as expected, NS1-HiBiT KDEL was not detected in supernatant samples, consistent with its expected retention in the ER.

To investigate whether the weak or undetectable expression of NS1 mutants in these Western blotting experiments could be attributed to inefficient transfection, lysates and supernatant samples from these experiments were also tested for intracellular and extracellular NS1 levels, respectively, via HiBiT luciferase reporter-based assays (Figure 4B). Intracellular NS1-associated luciferase activities were markedly higher than their corresponding extracellular values for the majority of these mutants, including V220D, A248V, T283A, L298W, C313S, I335T, and R336S, indicating that difficulties in detecting the expression of these mutants by anti-NS1 Western blotting cannot be attributed simply to low transfection efficiency (Figure 4B). DENV-2 NS1 Western blotting signals for each NS1 mutant, with the exception of T283A, C313S, and R336S, were also quantified before determining the extracellular-to-intracellular NS1 luminescence ratio (Figure 4C). The selected NS1 mutants displayed an impairment in the NS1 secretion efficiency, which is largely consistent with the results from the HiBiT-based luciferase reporter assays (Figure 1B,C).

Given difficulties in the detection of several NS1 mutant proteins using the conformation-specific anti-NS1 antibody, additional Western blotting studies using an anti-HiBiT peptide tag antibody were also performed. This revealed the ready detection of wildtype, KDEL-tagged, and mutant NS1-HiBiT proteins in intracellular lysate samples, although mutant constructs appeared to be expressed at lower levels than their wildtype and KDEL-tagged counterparts (Figure 4D). As expected, wildtype NS1-HiBiT was strongly detected in supernatants samples, while KDEL-tagged NS1-HiBiT was detected at an appreciably lower level in supernatant samples (Figure 4D). All NS1-HiBiT point mutants were detected at markedly reduced levels in supernatant samples (Figure 4D), with parallel analysis of HiBiT luciferase activity in lysate and supernatant samples largely reflecting the phenotypes that were observed via Western blot analysis (Figure 4E). A quantitative analysis of the NS1-HiBiT Western blot signals and an expression of these signals as normalised extracellular-to-intracellular NS1-HiBiT ratios was then performed (Figure 4F), and this largely confirmed the phenotypes that were observed in the original and Western-blot-parallel HiBiT luciferase assays (Figure 1C and Figure 4E). Surprisingly, in some instances, there were moderate inconsistencies between the strength of the HiBiT signals between Western blots and luciferase assays performed using the same samples (Figure 4A,B,D,E). It is possible that this is attributable to mutation-induced changes in the accessibility of the HiBiT peptide to its complementary LgBiT partner in the HiBiT luciferase assays. Taken together, these studies confirm the secretion-impaired phenotypes of all 10 NS1-HiBiT point mutants and indicate that at least some of these mutations may impact the ability of NS1 to be recognised by a conformation-specific anti-NS1 monoclonal antibody.

### 3.4. NS1 Secretion-Impaired Mutants and Wildtype NS1 Display Strong Colocalisation with the ER

NS1 is thought to participate in the membrane rearrangements of the ER to form VPs that house viral RNA replication events [7,9,21,22]. We, therefore, sought to investigate whether the secretion-defective NS1 mutants that we identified also displayed altered localisation with regard to the ER. To this end, we transiently transfected Huh-7.5 cells with NS1 expression constructs bearing their single point mutations, and assessed the localisation of NS1 with respect to the ER by high-resolution confocal imaging.

**Figure 5:**
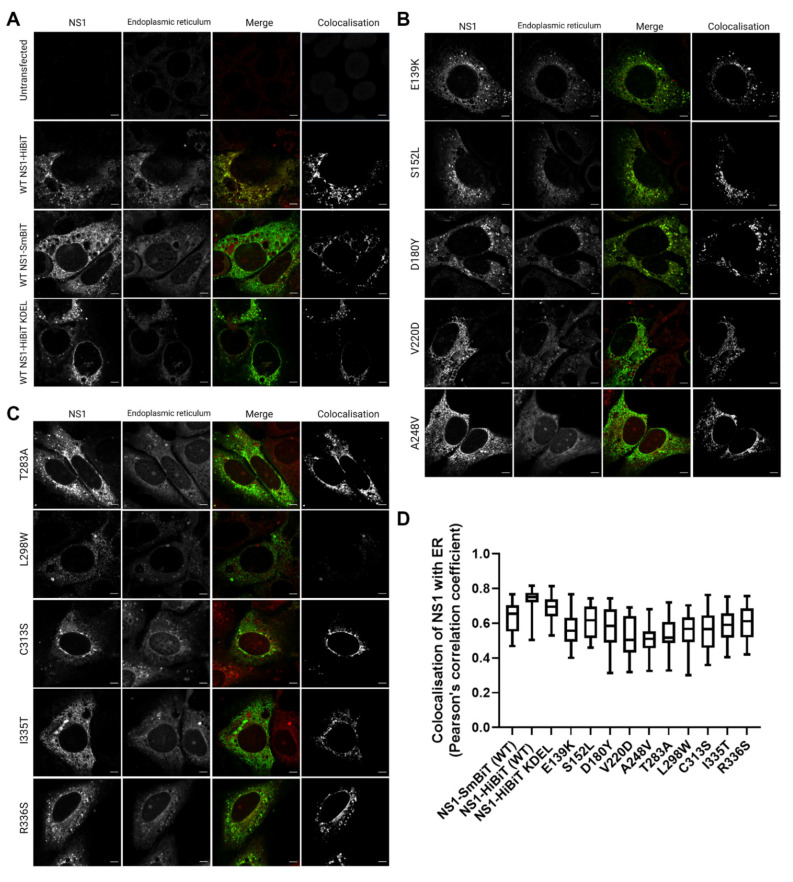
Identified secretion-impairing mutations within DENV NS1 do not impact its localisation to the ER. Huh-7.5 cells were transiently transfected with DENV NS1 expression constructs bearing respective NS1 secretion-impairing mutations and cultured overnight prior to fixation and fluorescent labelling. NS1 proteins (white, left column), endoplasmic reticulum (white, second column from the left), merged images (third column, with NS1 and ER staining pseudocolored green and red, respectively) and NS1-ER colocalised pixels (white, fourth column) are displayed in (**A**–**C**); (scale bars, 5 µM). (**D**) Colocalization between ER and NS1 signals (Pearson’s correlation coefficient) was measured for 20–25 cells per sample group. Box and whiskers plot indicates median values, 25th to 75th percentiles, maximum and minimum values (n = 20–25 cells/group).

All of the mutants, including the positive control for intracellular retention, NS1-HiBiT KDEL, and the negative control NS1-SmBiT utilised in the HiBiT luciferase reporter assays (Figure 1) displayed DENV-2 NS1 staining patterns that were highly similar to those of the wildtype NS1-HiBiT (Figure 5A–C). NS1 and an ER-specific fluorescent dye were strongly colocalised in all groups, as highlighted by a separate visualisation of colocalised signals, as shown (white; right panels) (Figure 5A–C). Additionally, colocalization analysis across a large number of cells revealed no significant differences between the groups (Figure 5D), indicating that none of the mutations appeared to disrupt the localisation of NS1 to the ER.

### 3.5. Effect of NS1 Secretion-Impairing Mutations on the DENV Replication Cycle

**Figure 6:**
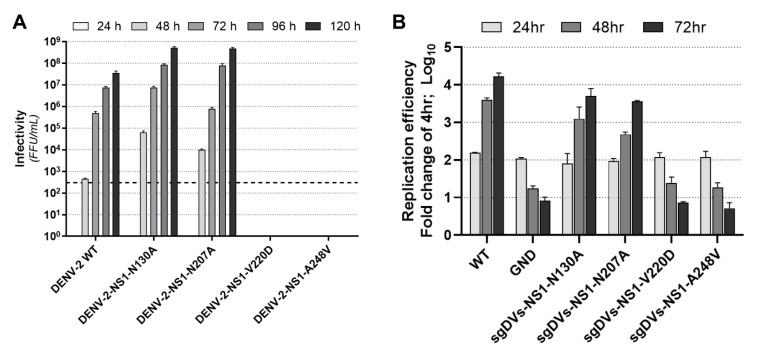
NS1 secretion-impairing mutations V220D and A248V prevent viral RNA replication and infectious virus production. (**A**) Infectious virus production by Huh-7.5 cells following transfection with wildtype (WT) DENV-2 RNA transcripts or derivatives containing NS1 secretion-impairing mutations (V220D or A248V) or N-linked glycosylation mutations (N130A or N207A), as indicated. Culture supernatants were harvested at indicated timepoints from 24 to 120 h post-transfection. Infectivity of viral particles within supernatants was determined by focus-forming unit (FFU) assay. Data are presented as means ± SD from three independent replicates. The dashed line indicates the limit of detection for the assay. (**B**) Replicative fitness of NS1 mutants in the context of a Renilla luciferase reporter encoding sub-genomic replicon construct (sgDVs-R2A). NS1 mutations in (**A**) were incorporated into sgDVs-R2A and in vitro transcribed RNA for each construct was transfected into Huh-7.5 cells. Luciferase activities were measured for lysates harvested at 4, 24, 48, and 72 h post-transfection. For each construct, raw luciferase values were expressed as a fold change relative to the average values for the 4 h timepoint (a measurement of input RNA) to determine replication efficiency. A replication-deficient lethal mutation within NS5 (GND) that disrupts NS5 RNA-dependent RNA polymerase activity was included as a negative control. Data are presented as means ± S.D from three independent replicates (n = 3).

Given their strong impact on NS1 secretion, we next investigated the influence of V220D and A248V mutations on the DENV replication cycle. We also chose to compare the effects of these mutations to those of two glycosylation mutations, N130A and N207A, which have been previously shown to impact NS1 secretion [30,31,50]. In vitro RNA transcripts of full-length constructs were transfected into Huh-7.5 cells, and culture supernatants were harvested at 24, 48, 72, 96, and 120 h post-transfection for measurement of viral infectivity by focus-forming unit (FFU) assays (Figure 6A). As shown, V220D and A248V mutations prevented infectious virus production across all timepoints. Interestingly, both N130A and N207A mutants were associated with marked increases in infectious virus production. In this particular context, a previous study showed that an N130Q mutation resulted in infectious virus titres that were similar to those of wildtype DENV [29].

To determine if the phenotypes observed for the V220D and A248V mutants were due to an impairment of viral RNA replication rather than a defect in infectious viral particle production, we also assessed the effects of these mutations on viral RNA replication. To this end, we incorporated these mutations into a sub-genomic replicon construct (sgDVs-R2A) that encodes a Renilla luciferase reporter [47]. Huh-7.5 cells were transfected with in vitro RNA transcripts for respective replicon constructs, and lysates were harvested at 4, 24, 48, and 72 h timepoints for measurement of luciferase activity as a readout of viral RNA replication/translation, compared to wildtype sgDVs-R2A and a replication-defective ‘GND’ control replicon (Figure 6B). While N130A and N207A mutant replicons displayed replication kinetics that were similar to that of the wildtype sub-genomic replicon, V220D and A248V mutants displayed steadily declining luciferase activities that reflected those of the replication-defective GND control. Together, this indicates that V220D and A248V mutations in NS1 prevent DENV-2 RNA replication, while mutations to N-glycosylation sites, N130A and N207A, were associated with unchanged viral RNA replication levels but moderated the elevated production of the infectious virus.

### 3.6. Utilisation of a Replication-Independent Expression System (pIRO) to Further Characterise Selected NS1 Secretion-Impaired Mutants

**Figure 7:**
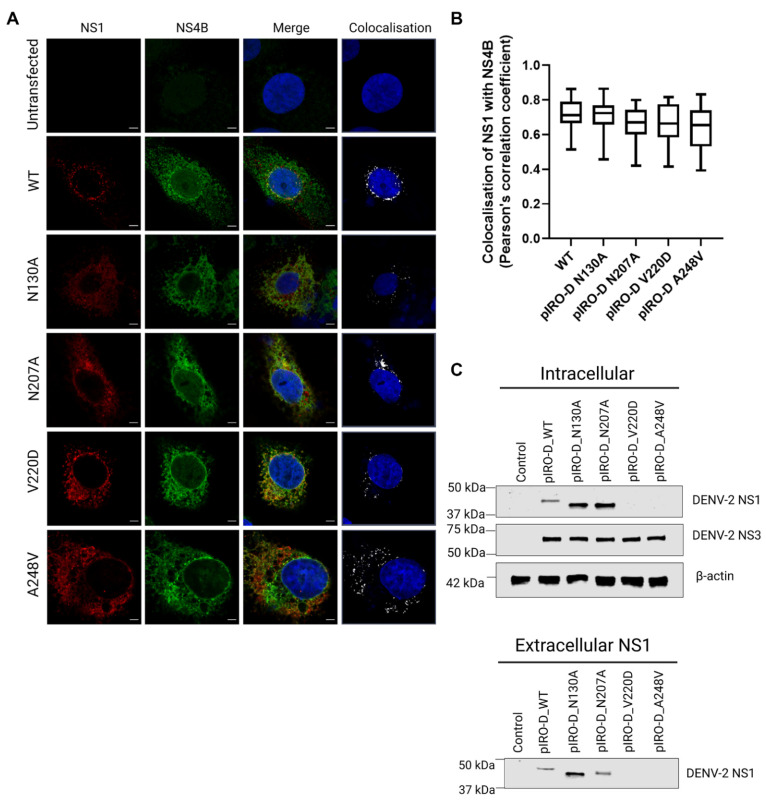
Characterisation of NS1 mutants using a replication-independent expression system (pIRO). (**A**) Near-confluent Huh-7.5 cells were transfected with pIRO expression constructs containing the indicated NS1 mutations (N130A/ N207A/ V220D/ A248V) and cultured overnight prior to fixation and immunolabelling. Confocal images of NS1 protein (red, left column), NS4B proteins (green, second column from the left), nuclei (blue), colocalization between NS1 and NS4B proteins in merge images (yellow, third column) or colocalization panels (white, fourth column) are depicted. Scale bars are 5 µm. (**B**) Quantification of colocalization between NS1 and NS4B for >30 identified cells/group. Box and whiskers plot indicates median values, 25th to 75th percentiles, maximum and minimum values (n = 30–32 cells/group). (**C**) Huh-7.5 were transiently transfected with the indicated pIRO expression constructs 48 h prior to collection of cell lysates and supernatants for Western blotting using antibodies NS1-, NS3- and β-actin, as indicated.

Given that the NS1 secretion-impaired mutants V220D and A248V were also replication-defective, we also further investigated the impact of these mutations using a recently established replication-independent expression system termed “pIRO” (plasmid-induced replication organelle formation) that induces the biogenesis of VPs that morphologically resemble conventional VPs generated in infected cells [51]. For this, we individually incorporated our mutations of interest, N130A, N207A, V220D, and A248V, into the pIRO expression construct using site-directed mutagenesis. To determine if these mutations induce any distinctive changes in the colocalization staining pattern between NS1 and NS4B, we transfected Huh-7.5 cells with the wildtype and mutant pIRO expression constructs 24 h prior to fixation, immunofluorescent staining of NS1 and NS4B, and high-resolution confocal imaging analyses (Figure 7A).

The pIRO-D mutants dictated similar NS1 and NS4B staining patterns in comparison to WT pIRO-D, although typical juxtanuclear NS1- and NS4B-positive foci were less frequently observed for N130A, V220D, and A248V mutants compared to wildtype. Colocalization analyses revealed no significant difference in NS1-NS4B colocalization (Figure 7B), suggesting that none of these mutations alter the association between NS1 and NS4B. However, NS1 in pIRO-D V220D and pIRO-D A248V lysates was not readily detected by Western blotting (Figure 7C), which was unexpected given our earlier results demonstrating that the same mutant NS1 proteins were detectable, albeit at low levels, when expressed in the context of NS1-HiBiT expression constructs (Figure 4A). In contrast, intracellular NS1 was strongly detected for WT, pIROD-N130A and pIROD-N207A. Extracellular NS1 was also detected in supernatants for WT, pIROD-N130A and pIROD-N207A, while NS3 was detected at a similar level in lysates of WT and all four pIRO-D mutants, indicating similar transfection efficiencies and NS protein expression levels across all pIRO-D transfected samples.

## 4. Discussion

Since its initial identification and characterisation in the 1970s, NS1 has been one of the most heavily studied flaviviral proteins. Over the years, it has emerged that this enigmatic viral non-structural protein is essential for viral RNA replication and infectious virus production, while the secreted form of NS1 is involved in a variety of extracellular functions strongly linked with flaviviral disease pathogenesis. Despite this, the exact molecular features of NS1 that are essential for its secretion remain unclear.

In this study, we applied random point mutagenesis, sensitive luciferase-based expression assays, and molecular virology approaches to identify NS1 residues that are required for NS1 secretion, and subsequently investigated the impact of two of these identified mutations on aspects of the DENV life cycle. There are several advantages and disadvantages of our chosen random point mutagenesis-coupled HiBiT luciferase assay screening approach in which we only sequenced clones with a >50% reduction in NS1 secretion efficiency. While this approach enables a simple, rapid, unbiased, and relatively inexpensive identification of secretion-defective NS1 mutants, despite attempts to carefully control PCR-based mutagenesis rates to result in one mutation per clone, many of the secretion-defective NS1 clones contained several or multiple mutations that could not be disentangled from one another without subsequent site-directed mutagenesis and follow-up experimentation. Conversely, as we chose not to sequence and characterise clones that displayed near-normal NS1 secretion, it is arguable that we missed the opportunity to identify residues and regions of NS1 that are not involved in its secretion. Furthermore, while our NS1-HiBiT expression approach enabled a simple and relatively rapid screening of approximately 170 clones for changes in the efficiency of NS1 secretion, mutations that specifically disrupted NS1 secretion could not be readily distinguished from mutations that indirectly affected NS1 secretion, as might be the result of NS1 mis-folding or failure to dimerise. For these given reasons, it remains important to consider that there are several potential reasons that a given NS1 mutant may display altered secretion and that detailed analysis in the context of a full-length infectious virus is essential to fully appreciate the underlying impact of a given mutation.

Relevant to our study, Plaszczyca et al. (2019) identified an NS1 secretion-impairing mutation, D136A, within the Wing domain, as well as a series of NS1 secretion-impairing mutations, W311A, P319A, P320A, E334A, and R336A within the C-terminal β-ladder domain [44]. Consistent with that study, our analyses also identified Arg-336 as an important determinant of NS1 secretion as its mutation (R336S) similarly inhibited NS1 secretion in our HiBiT-tagged expression system. Of note, Arg-336 has also been shown to be essential for viral RNA replication, indicating that this residue is important for multiple functions of NS1, and perhaps correct NS1 maturation and/or interactions may be disrupted by its mutation [22]. It is also noteworthy that several of the NS1 secretion-defective mutations that we identified, E139K, C313S, and I335T, mapped in close proximity to NS1 secretion-defective mutations that were identified by Plaszczyca et al.; namely, D136A, W311A, E334A and R336A [44]. This supports the interpretation that these sub-domains are important for NS1 secretion, and that the solvent-exposed nature of these sites within the C-terminus of the β-ladder and, to a lesser extent, the Wing domain may mediate NS1 interactions that are important for its secretion.

Intriguingly, NS1 protein was not readily detected in transfected cell lysates or supernatants for several NS1 mutants when analysed by Western blotting using a conformation-specific anti-NS1 antibody. This could not be simply attributed to inefficient transfection, as many of the mutant NS1 proteins that could not be detected by Western blotting nonetheless displayed robust HiBiT luciferase assay values in the same lysates. The inability to detect NS1 mutants in several lysate and supernatant samples using the conformation-specific anti-NS1 antibody could be potentially attributed to mutation-mediated disruption of residues that are involved in the binding of this antibody or impairment of correct NS1 folding and/or maturation, such that the protein cannot be recognised by the antibody. In support of this possibility, Western blot studies using an anti-HiBiT peptide tag antibody revealed ready detection of all mutant proteins and confirmed the secretion-impaired phenotypes of these mutants that were indicated by HiBiT luciferase assays.

Interestingly, the mutations that altered NS1 secretion efficiency did not cause dramatic changes in the localisation of NS1 protein or its colocalization with the ER. This indicates that the impaired secretion of these NS1 mutant proteins cannot be easily attributed to the gross mislocalisation of the protein, although more detailed analyses are required to determine whether these mutations induce more subtle changes in NS1 localisation. In regard to the discrepancies between the detection of many of these NS1 mutant proteins by immunofluorescence but not Western blotting using the same antibody, it is possible that the mutant proteins are much more sensitive than wildtype NS1 to sample the processing steps involved in Western blotting, such as lysis in strong detergents and boiling, as compared to less destructive steps involved in immunofluorescent labelling. Future studies of the localization of these NS1 mutant proteins using anti-NS1 antibodies that recognize linear epitopes may help to clarify whether the secretion-impairing mutations indeed alter NS1 localization within cells.

Given that N-glycans within DENV NS1 are likely involved in NS1 secretion and NS1 dimer stability, we decided to include two N-glycosylation mutants, N130A and N207A, in our studies [29,30,31]. Both V220D and A248V mutants did not support DENV RNA replication or infectious virus production, indicating that these relatively highly conserved residues are critical to multiple NS1 functions. In contrast, both N-glycosylation mutants displayed RNA replication and virus particle production levels that were similar to those of the wildtype virus, which is largely consistent with previous reports [29,50].

Taken together, our findings suggest that mutating the relatively highly conserved residues V220 and A248 may lead to conformational changes in the NS1 structure resulting in its inherent instability or improper folding. For example, the introduction of a charged aspartate residue in place of the hydrophobic valine residue in the V220D mutant may disrupt intramolecular or intermolecular interactions within this ‘spaghetti loop’ region of the β-ladder to disrupt folding and conformation. In contrast, the conservative A248V substitution in the same domain is perhaps less likely to greatly alter NS1 conformation. It was initially considered that these mutations may cause NS1 mislocalisation, thereby disrupting viral replication and viral particle production in the process. However, heterologously expressed NS1-V220D and NS1-A248V displayed localisation profiles and colocalization with the endoplasmic reticulum that were similar to those of heterologously expressed wildtype NS1. Additionally, both NS1 mutants colocalised with NS4B when expressed via T7 RNA polymerase in the context of an NS1-NS5 polyprotein, which suggests that their interaction with NS4B is not overtly affected or compromised. This possibility was investigated, given the previous demonstration that NS1 may play a role in the stabilisation of membranous viral replication complexes, via its interaction with NS4B [19,20,22].

In summary, this study has demonstrated the utility of combining the random mutagenesis of NS1 and the HiBiT luminescent peptide tagging system in the rapid and simple identification of NS1 secretion-impairing mutants. Using this system, we have identified ten point mutations associated with an impairment in NS1 secretion, with two mutations (V220D and A248V) shown to also be critical for viral RNA replication. We suggest that future identification of mutations that prevent NS1 secretion but do not markedly attenuate viral fitness will reveal new details about dengue virus biology and may represent a promising strategy towards the development of an attenuated vaccine to combat DENV.

## Data Availability

Original data files are available on request.

## Supplementary Materials

The following supporting information can be downloaded at: https://www.mdpi.com/article/10.3390/v15051102/s1. Table S1: Antibodies and Dyes [52]; Table S2: Oligonucleotide sequences (5’ to 3’).
