# Supplementary material for: Identification of Key Residues in Dengue Virus NS1 Protein That Are Essential for Its Secretion
Source: Viruses. 2023 Apr 30;15(5):1102. doi: 10.3390/v15051102 (PMC10221731; doi:10.3390/v15051102)
Supplement: Supplementary file 1 [file viruses-15-01102-s001.zip › Supplementary Table S1 Antibodies and Dyes.pdf]

Supplementary Table S1. Antibodies and Dyes

| Reagent | | Manufacturer/ source | Identifier |
|---|---|---|---|
| Antibodies | Mouse anti-NS1 '4G4' | Mozzy Mabs | MMABS 4G4* |
| | DENV NS3 (GT2811) | GeneTex | GTX629477 |
| | DENV NS4B | GeneTex | GTX12450 |
| | Mouse anti-capsid '6F3.1' | Prof. John Aaskov (QUT,Brisbane, Australia) | N/A |
| | Mouse anti-HiBiT (Clone 30E5) | Promega | Early Access |
| | Monoclonal mouse anti β-actin | Sigma Aldrich | A5441 |
| | IR®Dye 800CW goat-anti mouse | LI-COR | 926-32210 |
| | Alexa Fluor 488 goat-anti mouse | Thermo Fisher Scientific | A11001 |
| | Alexa Fluor 488 goat anti-rabbit | Thermo Fisher Scientific | A11008 |
| | Alexa Fluor 555 goat anti-mouse | Thermo Fisher Scientific | A21422 |
| Dyes | DAPI | Sigma Aldrich | D9542-5MG |
| | IraZolve-ER Blue® | REZOLVE Scientific, Australia | 1101026 |

*Anti-NS1 mAb 4G4 was raised against immunopurified NS1 prepared from MVEV-infected cells [52]. The exact epitope that is recognises is unknown.
