# Supplementary material for: Identification of Key Residues in Dengue Virus NS1 Protein That Are Essential for Its Secretion
Source: Viruses. 2023 Apr 30;15(5):1102. doi: 10.3390/v15051102 (PMC10221731; doi:10.3390/v15051102)
Supplement: Supplementary file 1 [file viruses-15-01102-s001.zip › Supplementary Table S2 Oligonucleotide Sequences.pdf]

Supplementary Table S2. Oligonucleotide sequences (5′ to 3′)

MluI ext FWD	TTTGTGGAATCCGCTCAGTAACGCGTCT
KasI ext REV	CCTATGTCATCCGTCATAGTGGCGCCTACCATAACC
NS1 N130A FWD	GCTCTCTACAGAGTCTCAT**GC**CCAGACCTTTCTCATTGATGGCCC
NS1 N130A REV	CCATCAATGAGAAAGGTCTGG**GC**ATGAGACTCTGTAGAGAGCAT
NS1 N207A FWD	TGGATAGAAAGTGCACTC**GC**TGACACATGGAAGATAGAGAAAGCCTC
NS1 N207A REV	TCTCTATCTTCCATGTGTCA**GC**GAGTGCACTTTCTATCCAATAACC
NS1 V220D FWD	AAAGCCTCTTTCATTGAAG**A**TAAAAACTGCCACTGGCCAA
NS1 V220D REV	TGGCCAGTGGCAGTTTTTA**T**CTTCAATGAAAGAGGCTTTCT
NS1 A248V FWD	ATAATTCCAAAGAATCTCG**T**TGGACCAGTGTCTCAACACAACTA
NS1 A248V REV	GTGTTGAGACACTGGTCCA**A**CGAGATTCTTTGGAATTATCATCT
pLenti6 NS1 FWD	TAGAAGACACCGACTCTAGACCACCATGAGCACCTCACTGTCTGTG
pLenti6 NS1 REV	GCCAGCCGCTCACTGATCCCCCAGCTGTGACCAAGGAGTTG
HiBiT TOP FWD	CTCGAGGGATCAGTGAGCGGCTGGCGGCTGTTCAAGAAGATTAGCTAGA
HiBiT BOT REV	CGCGTCTAGCTAATCTTCTTGAACAGCCGCCAGCCGCTCACTGATCCC
